# Experience in the diagnosis and treatment of venous adventitial cystic disease: a case series and literature review

**DOI:** 10.3389/fsurg.2026.1750548

**Published:** 2026-05-07

**Authors:** Jianhuang Zhuang, Songlin Guo, Lei Wang, Longlong Zheng, Meilin Liu, Zhuohang Wu, Xiaoqiang Zhang, Zhang Zhang

**Affiliations:** Department of Intervention Radiology and Vascular Surgery, Tangdu Hospital, Fourth Military Medical University, Xi'an, China

**Keywords:** case report, common femoral vein, lymphatic malformation, open surgery, venous adventitial cystic disease

## Abstract

Venous adventitial cystic disease (VACD) is a rare condition. We describe two cases of unilateral lower-limb swelling due to a cystic mass compressing the common femoral vein (CFV) treated within a single vascular surgery unit. The first case was misdiagnosed as a lymphatic cyst preoperatively on both computed tomography and Doppler ultrasound. However, intraoperative findings suggested VACD, which was confirmed histopathologically. The second patient received the correct preoperative and postoperative VACD diagnoses. Both patients were successfully treated with surgical resection, with no complications during the postoperative follow-up.

## 1 Introduction

Venous adventitial cystic disease (VACD) is a rare venous occurrence, accounting for 5.3%–9.3% of all adventitial cystic disease cases (1). It is characterized by the formation of gelatinous mucoid cystic cavities in the adventitia of vessels near joints, which contain fluid similar to synovial fluid (2). Owing to the varying locations of vascular cysts and the degree of venous lumen obstruction, the clinical manifestations are non-specific and similar to those of other venous malformations, often leading to misdiagnosis as deep vein thrombosis, varicose veins, venous aneurysms, or lymphatic diseases (3). Currently, there is no consensus on the etiology, pathogenesis, or treatment protocols for VACD. This article summarizes and analyzes the diagnosis and treatment processes of VACD at our center and reviews relevant published literature. This study aimed to summarize the imaging misdiagnosis patterns in VACD and verify the effectiveness of cystectomy.

## 2 Case report

### 2.1 Case 1

A 41-year-old otherwise healthy man presented to our outpatient vascular clinic with swelling in his left leg. His symptoms gradually developed over 2 weeks, with no history of trauma or strenuous activity.

**Figure 1:**
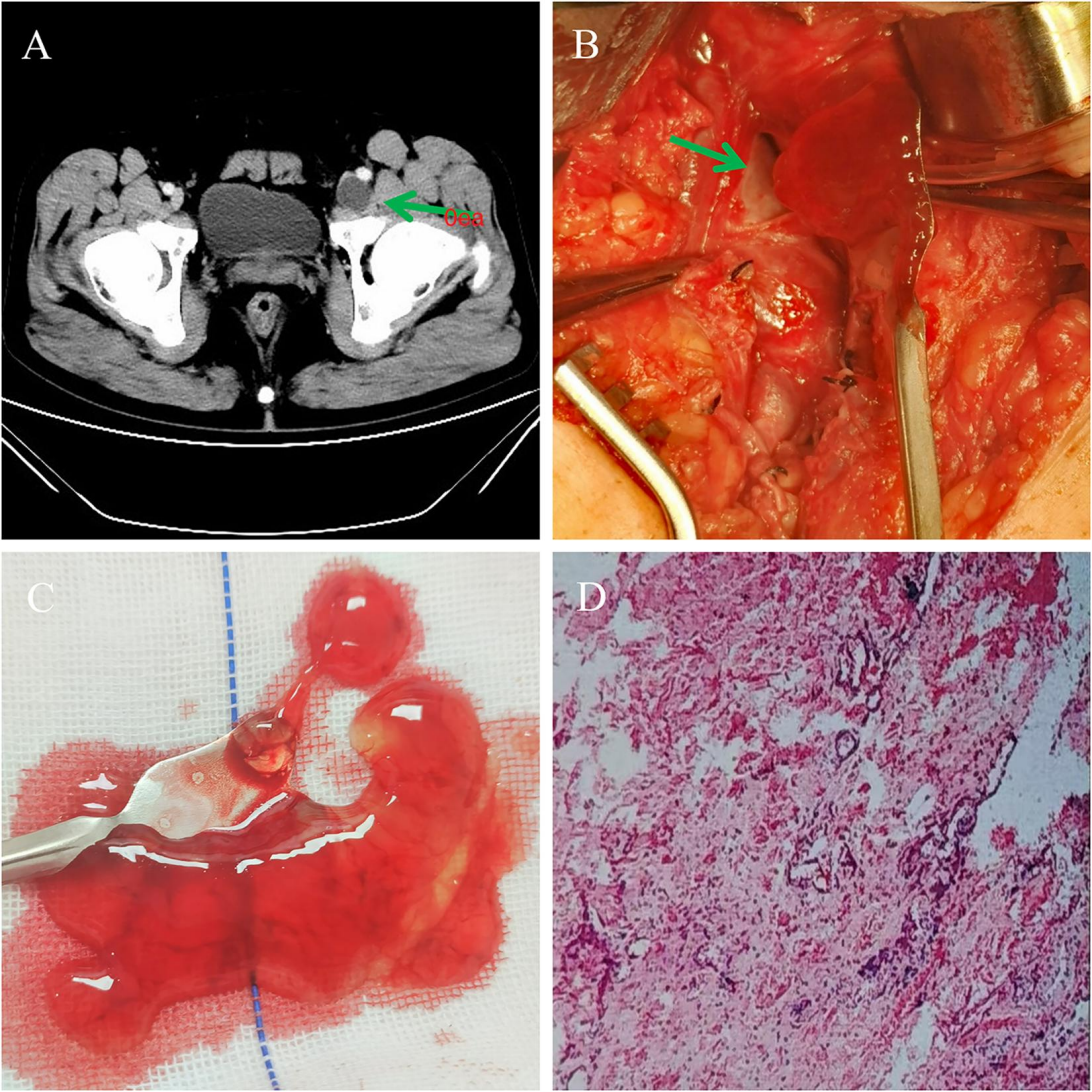
Case 1 findings. **(A)** CT revealed a round, hypoechoic, low-density lesion medial to the left CFV in the left inguinal region (arrow). **(B)** The gelatinous content of the adventitial cyst caused severe stenosis. Upon dissection of the cysts, no connections were found with the vessel lumen (arrow). **(C)** The cyst cavity was filled with a pale-yellow, jelly-like fluid. **(D)** Microscopic image of a section stained with hematoxylin-eosin (original magnification: ×100). The cyst wall consisted of fibrovascular tissue with fibrous proliferation and hyaline degeneration, while the cyst contents included a few red blood cells and occasional monocytes, consistent with VACD.

Physical examination revealed no palpable masses in the left inguinal region. The soft tissue of the left lower limb showed non-pitting edema. Swelling was observed in the entire limb, and the left leg was 5.0 cm wider than the right leg. The skin temperatures of both lower limbs were normal, and the pulses of the major arteries on both sides were palpable. Duplex ultrasound demonstrated a hypoechoic area measuring 1.98 cm × 3.74 cm medial to the left common femoral vein (CFV), with clear boundaries and a regular shape, and the left CFV was compressed, with accelerated blood flow. Computed tomography (CT) revealed a round, hypoechoic, low-density lesion measuring approximately 2.4 cm × 2.1 cm × 2.9 cm medial to the left CFV in the left inguinal region. No significant enhancement was observed on contrast-enhanced scans, and a lymphatic cyst was considered a possible diagnosis (Figure 1A). Although a lymphatic cyst was suspected at that time, the definitive diagnosis remained undetermined, and the differential diagnosis included other etiologies such as venous aneurysm and VACD.

Considering the severity of his symptoms and his acceptable surgical risk, we offered open surgical repair. The common femoral artery and vein were exposed through a longitudinal incision. Intraoperative exploration confirmed the femoral artery and lymphatic vessels to be structurally intact and morphologically normal. This assessment was based on direct visualization of smooth vessel walls; the absence of dilation, stenosis, or abnormal masses; and the preservation of the typical anatomical course. No evidence of cystic involvement or pathological changes was observed in these structures. A cystic adventitial swelling measuring approximately 3.0 cm × 2.0 cm was observed on the posteromedial aspect of the CFV. The stenosis appeared to be secondary to compression by the cystic structures within the venous wall (Figure 1B). Intraoperative Doppler ultrasound was used to confirm venous patency before dissection. Upon incision of the cyst wall, the cyst cavity was filled with a pale-yellow jelly-like fluid (Figure 1C). After draining the cystic fluid, further exploration revealed no connection between the cystic cavity and the hip joint. The cyst was unilocular and existed independently. Redundant cyst walls were meticulously trimmed, whereas those adhering to the venous adventitia were preserved to avoid iatrogenic vascular injury. The wound was closed layer by layer, and a local compression dressing was applied. The postoperative pathology revealed fibrovascular tissue with fibrous proliferation and hyaline degeneration in the cyst wall. The cyst contents included a few red blood cells and occasional monocytes. These findings were confirmed by hematoxylin and eosin staining and were compatible with VACD (Figure 1D). Based on the intraoperative findings and postoperative pathology, the diagnosis was corrected to cystic adventitial disease of the left femoral vein. Postoperatively, the patient underwent anticoagulation therapy with enoxaparin sodium (5,000 IU, s.c., q12), and the swelling of the left lower limb subsided. The patient was discharged 8 days after recovery. A follow-up vascular ultrasound 1 month later demonstrated patent blood flow in the left femoral vein, and no recurrence of symptoms was observed during the 1-year follow-up.

### 2.2 Case 2

**Figure 2:**
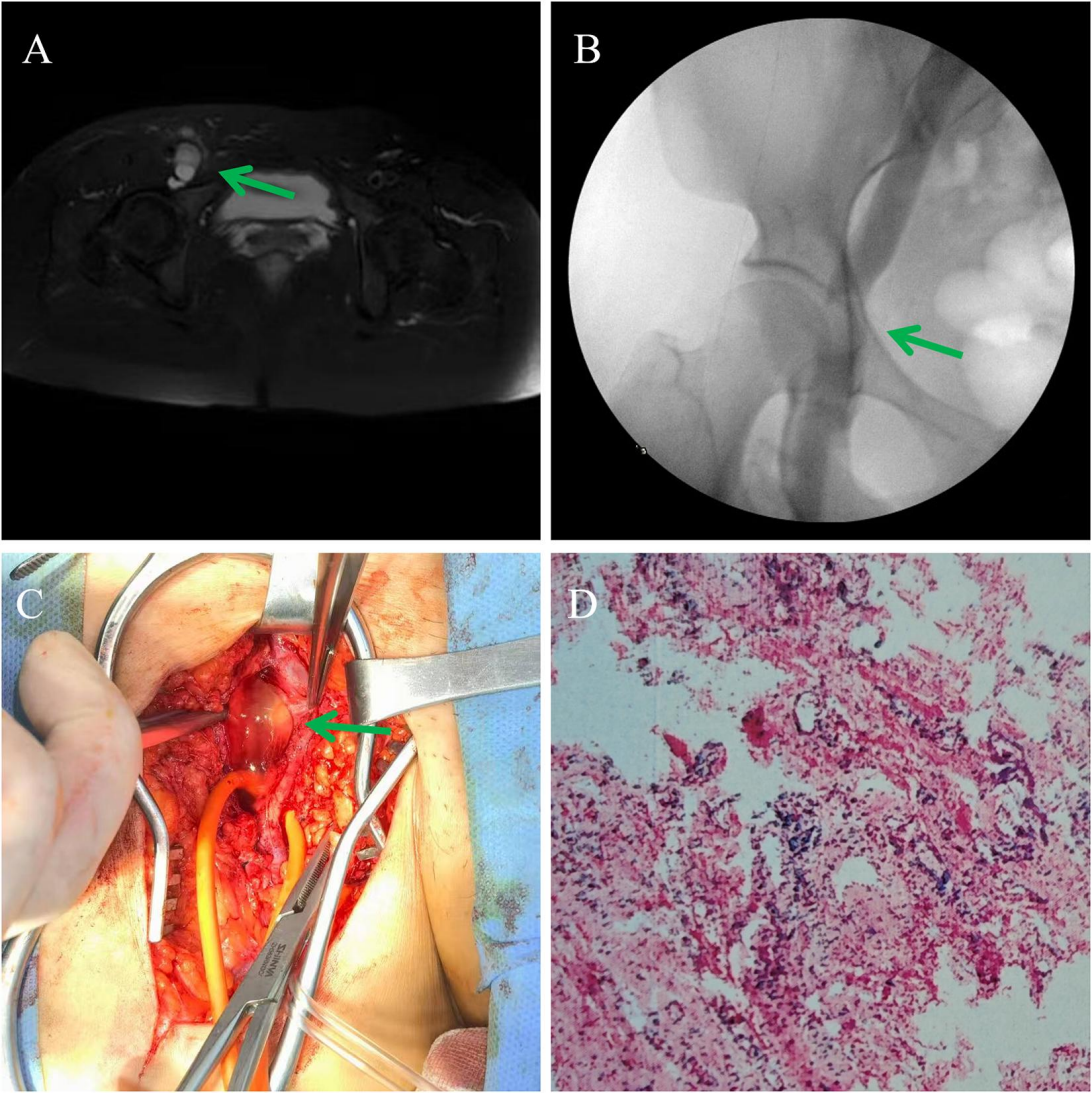
Case 2 findings. **(A)** MRI T2-weighted Dixon sequence demonstrated a hyperintense mass with a thin isointense capsule surrounding the right CFV (arrow). **(B)** Venography showed a mass-like lesion, closely related to the blood vessel and pressing on the right CFV (arrow). **(C)** Operative view showed mucoid fluid, which was filled in the cystic wall. **(D)** Microscopic image of a section stained with hematoxylin-eosin (original magnification: ×100). Histological examination of the cystic mass confirmed VACD.

A 36-year-old woman presented to the vascular surgery outpatient department with a 2-month history of worsening swelling in her right leg that affected her walking. The patient had no history of comorbidities, trauma, or surgery. Physical examination revealed swelling in the right leg without tenderness and a palpable mass in the right inguinal region. The popliteal and pedal pulses in the right leg were normal. Doppler ultrasound examination detected a cystic mass, approximately 2.5 cm × 2.7 cm × 2.4 cm in size, with clear boundaries closely related to the right CFV. Magnetic resonance imaging (MRI) was performed to obtain additional information and to exclude other potential causes of vessel obstruction, such as hemangioma or exogenous pressure. MRI scans revealed multiple isolated and exogenously grown cystic masses in the right CFV segment, and the collateral circulation was partially dilatated (Figure 2A). Venographic examination of the right leg revealed a marked compression of the femoral vein (Figure 2B). Therefore, a clinical diagnosis of VACD was proposed and surgical excision was performed. Although VACD was suspected at that time, the definitive diagnosis remained undetermined, and the differential diagnosis included other etiologies such as a benign venous tumor, lymphatic malformation, or synovial cyst. Under total intravenous anesthesia, the right CFV, femoral artery, and lymphatic vessels were adequately exposed and explored. Intraoperative exploration confirmed the femoral artery and lymphatic vessels to be structurally intact and morphologically normal. This assessment was based on direct visualization of smooth vessel walls; the absence of dilation, stenosis, or abnormal masses; and the preservation of the typical anatomical course. No evidence of cystic involvement or pathological changes was observed in these structures. The entire right CFV was dissected and controlled. Four unconnected adventitial cysts of varying sizes were visible on the posterior aspect of the femoral vein, with the largest measuring approximately 2 cm × 3 cm. The cysts were incised to remove the fluid, the redundant cyst walls were meticulously trimmed, and those adhering to the venous adventitia were deliberately preserved to avoid iatrogenic vascular injury. The cyst was drained of its fluid, and further exploration revealed no connection between the cyst cavity and the hip joint (Figure 2C). Histological examination of the dissected cyst clearly demonstrated a cyst wall lined by dense fibrous connective tissue, consistent with a benign cyst, which confirmed VACD (Figure 2D). Postoperatively, the patient underwent anticoagulation therapy with enoxaparin sodium (5,000 IU, s.c., q12), and the limb swelling resolved 1 week later. At the 3-month follow-up, the leg swelling had resolved and the CFV was patent on vascular ultrasound imaging without a mass effect. No recurrence of symptoms was observed during the 6-month follow-up.

## 3 Literature review

### 3.1 Epidemiology and clinical features

VACD is a rare, benign vascular disease that is more common in middle-aged men with a mean age of 47 ± 14 years (range 5–75 years) and typically occurs on the left side (4). Bascone et al. (5) reviewed 45 cases of VACD reported over 50 years (1963–2016) and found that the femoral vein was the most commonly involved site (56%). However, VACD of the femoral vein is extremely rare, with only 44 cases reported between 1947 and 2020 (6). Symptoms depend on the cyst location and the degree of vein obstruction. However, a correct preoperative diagnosis is seldom established owing to the rarity of the condition and its non-specific presentation.

### 3.2 Etiology and pathophysiology

The etiology of VACD remains debated, with two prevailing hypotheses, i.e., the developmental theory (embryonic mesenchymal cell migration into the venous adventitia) and the synovial theory (synovial fluid leakage from adjacent joints) (3, 6). While the developmental theory is widely cited, Cai and Loa reported synovial anomalies in 17% of patients with ACD (7). Notably, whether these arterial-derived theories fully apply to venous variants remains to be validated, given the paucity of VACD cases (8–10). Histopathologically, the cysts may be unilocular or multilocular and contain an eosinophilic mucoid gel containing mucin and glycosaminoglycans. This mucoid gel destroys the elastic tissue between the tunica media and tunica adventitia of the vein and is replaced by fibrous connective tissue (3). Macroscopically, the cystic cavity is filled with yellow or transparent jelly-like material (11). Further studies are needed to elucidate the underlying molecular and cellular mechanisms of VACD, which could lead to the development of new therapies.

### 3.3 Imaging diagnosis

An accurate diagnosis of VACD begins with imaging studies, including Doppler ultrasound, CT venography, contrast-enhanced CT, and MRI (5). Doppler ultrasound is the preferred imaging modality for identifying cysts on the vascular wall, which appear as typical hypoechoic cystic lesions with or without septations. CT venography delineates the location and extent of venous flow obstruction and directly visualizes the characteristic hourglass-, scimitar-, or fan-shaped appearance caused by extraluminal compression. CT and MRI can localize the lesion to the vascular wall and identify the cysts causing the extraluminal compression. Contrast-enhanced CT shows low-density cystic lesions on the vascular wall, while MRI depicts the cysts as a homogeneous low signal on T1WI and a multilocular high signal adjacent to the vessel on T2WI (10, 12, 13). However, the clinical symptoms of lymphatic malformation and VACD in the inguinal region are caused by a mass effect with similar imaging manifestations, suggesting a perivascular hypoechoic cyst (14–16).

VACD can be easily confused with extrinsic lymphatic malformation compression and joint-related cysts, which interfere with an accurate preoperative diagnosis. Atamne et al. (17) emphasized the need to maintain a VACD diagnosis during imaging studies until it is confirmed by surgical findings (cysts sharing a common wall with the vessel) and pathological results (epithelial lining with synovial cells). Preoperative imaging is critical for detecting any connections between the cyst and the joint cavity and its location, allowing appropriate surgical planning and reducing recurrence. Therefore, while imaging provides ancillary diagnostic value, surgical exploration and pathology remain the gold standard.

### 3.4 Treatment modalities

Case reports and small series constitute the available treatment literature (16). Surgical approaches include open- or image-guided percutaneous cyst aspiration, stent placement, and cyst excision (with or without vascular reconstruction) (17). Lim et al. (18) reported that different surgical strategies have varying rates of symptom control and recurrence. Cyst excision is a commonly used treatment for VACD, with Biggs et al. (4) reporting a recurrence rate of 7% after cyst excision or combined vein resection. However, recurrence is common when the wall of the cyst is not completely excised or the joint connection is not obliterated. Cyst aspiration has significant immediate benefits, but the incomplete evacuation of cysts because of high viscosity results in a high recurrence rate (83.3%) (19). This may be due to the persistence of the secretory components of the cysts despite aspiration or the fact that aspiration of mucoid contents is not technically feasible with the fine-gauge needle needed to avoid excessive trauma to the vessel. Endovascular stent placement does not address the root cause of compression, and stents spanning the joints with low venous flow are prone to in-stent thrombosis. Multiple studies have noted higher rates of recurrence with endovascular techniques, perhaps due to the inability to address joint connections if present (2–5). The surgical removal of the intracavity cyst, followed by venous angioplasty, theoretically, should relieve the blood vessel from obstruction. Comparative studies are needed to evaluate the efficacy of different surgical approaches and minimally invasive techniques, as well as the role of adjuvant therapies.

## 4 Discussion

The present study summarizes two cases of VACD treated at a single vascular center, highlighting diagnostic challenges and the effectiveness of open surgical resection. In Case 1, preoperative CT and Doppler ultrasound misdiagnosed the lesion as a lymphatic cyst, whereas intraoperative findings and postoperative pathology confirmed VACD. In Case 2, MRI and venography enabled a correct preoperative diagnosis. Both patients underwent successful open cyst excision with uneventful recovery and no recurrence during follow-up.

The rarity and non-specific symptoms of VACD contribute to frequent misdiagnosis, as seen in Case 1. Imaging studies help identify cystic lesions and venous compression but cannot reliably distinguish VACD from lymphatic malformations. The misdiagnosis in Case 1 underscores the necessity of surgical verification in cystic venous pathologies, while Case 2 exemplifies the critical role of histopathology in differentiating VACD from lymphatic malformations, despite overlapping imaging features. The development of more sensitive and specific imaging modalities and biomarkers may improve the early diagnosis of VACD, reducing the risk of misdiagnosis and delayed treatment.

Surgical exploration with complete cyst resection is the optimal management strategy. In Case 1 and Case 2, given the patient's refusal to undergo autologous vein or prosthetic graft replacement of the involved venous segment, a cyst wall-trimming approach with conservative preservation of the adventitia-adherent cyst walls was implemented to minimize vascular intervention. Larger cysts were aspirated to create a working space, allowing for the precise removal of smaller, adjacent cysts without damaging the venous wall. To avoid venous injury, the redundant cyst walls were meticulously trimmed, and those adhering to the venous adventitia were deliberately preserved to avoid iatrogenic vascular injury. Considering the potential for vascular wall injury, postoperative enoxaparin sodium was administered to prevent thrombosis. No intraoperative or postoperative complications occurred. No venous thrombosis, hematoma, or wound infection was observed. Both patients had good outcomes with no recurrence during the follow-up. A potential limitation of this study is the discrepancy in follow-up duration between the two cases, with 12 months for Case 1 and 6 months for Case 2. This difference limits the direct comparability of long-term treatment outcomes. Future studies with larger sample sizes and uniform follow-up periods are needed to further validate the long-term efficacy of surgical resection for VACD.

## 5 Conclusions

The unclear etiology and non-specific clinical manifestations of VACD lead to its misdiagnosis and missed diagnosis. VACD should be highly suspected when patients present with spontaneous unilateral limb swelling and imaging studies reveal hypoechoic cysts around the vessels. Imaging provides ancillary diagnostic value, and intraoperative exploration and pathology remain the diagnostic gold standard. Once confirmed, cyst excision is the treatment of choice, removing the cyst wall as completely as possible. If the cyst wall adheres to the vein, the involved vein should be removed to reduce the risk of recurrence, and regular follow-up with physical and imaging examinations is recommended.

## Data Availability

The original contributions presented in the study are included in the article/Supplementary Material, further inquiries can be directed to the corresponding author.

## References

[1] Hashimoto M Tamate Y Sato H Murakami A Yanagawa N. Long-term outcome of partial resection in venous adventitial cystic disease. J Vasc Surg Cases Innov Tech*.* (2021) 7(3):382–5. 10.1016/j.jvscit.2021.04.022 34278063 PMC8261535

[2] Bae M Huh U Lee CW Kim JW. Venous adventitial cystic disease is a very rare disease that can cause deep vein thrombosis: a case report. World J Clin Cases*.* (2023) 11(34):8170–5. 10.12998/wjcc.v11.i34.8170 38130778 PMC10731187

[3] Tan R Tosenovsky P. Venous adventitial cyst mimicking a persistent femoral deep venous thrombosis. Ann Vasc Surg. (2021) 73:511–4. 10.1016/j.avsg.2020.12.014 33515660

[4] Biggs JH Kalra M Skinner JA DeMartino RR. Adventitial cystic disease of the common femoral vein: an unusual cause of lower extremity swelling and review of the literature. J Vasc Surg Cases Innov Tech*.* (2021) 7(4):610–6. 10.1016/j.jvscit.2021.06.015 34746527 PMC8551497

[5] Bascone C Iqbal M Narh-Martey P Szuchmacher M Cicchillo M Krishnasastry KV. Venous adventitial cystic disease: a review of 45 cases treated since 1963. Int J Vasc Med. (2016) 2016:5287697. 10.1155/2016/5287697 27885342 PMC5112310

[6] Kunimoto H Honda K Nakamura R Nishimura Y. Adventitial cystic disease of the femoral vein accompanied by deep vein thrombosis. Interact Cardiovasc Thorac Surg*.* (2021) 33(1):142–4. 10.1093/icvts/ivab040 33575741 PMC8691554

[7] Cai TY Loa J. Multimodal imaging demonstrating adventitial cystic disease of the common femoral vein. ANZ J Surg*.* (2021) 91(5):E330–1. 10.1111/ans.16363 32997427

[8] Desy NM Spinner RJ. The etiology and management of cystic adventitial disease. J Vasc Surg*.* (2014) 60(1):235–45, 245.e1–11. 10.1016/j.jvs.2014.04.014 24970659

[9] Iwasaki A Furukawa K Nakamura E Nakamura K. Adventitial cystic disease of the common femoral vein misdiagnosed as deep vein thrombosis. Interact Cardiovasc Thorac Surg*.* (2018) 27(2):312–3. 10.1093/icvts/ivy052 29509904

[10] Min SK Han A Min S Park YJ. Inconsistent use of terminology and different treatment outcomes of venous adventitial cystic disease: a proposal for reporting standards. Vasc Specialist Int. (2020) 36:57–65. 10.5758/vsi.200029 32611837 PMC7333088

[11] Flynn D Tesar J Peden S Quinn S Kruger A Jenkins J. Venous cystic adventitial disease: to cure or manage? A case series. BMJ Case Rep*.* (2022) 15(1):e247813. 10.1136/bcr-2021-247813 35027391 PMC8762143

[12] Tinelli G Montanari F Minelli F De Nigris F Sica S Tshomba Y. Long-term follow-up of adventitial cyst surgical excision in external iliac vein. J Vasc Surg Cases Innov Tech*.* (2020) 6(3):320–3. 10.1016/j.jvscit.2020.04.008 33367187 PMC7748988

[13] Guo F Guo Y. Cystic adventitial disease of the common femoral vein: a case report. Vascular*.* (2020) 28(4):489–93. 10.1177/1708538120915297 32281495

[14] Kulungowski AM Patel M. Lymphatic malformations. Semin Pediatr Surg. (2020) 29(5):150971. 10.1016/j.sempedsurg.2020.150971 33069296

[15] Vásquez-Castillo AC Moreira J Castillo G Hamam JE Masri MM. Inguinal intranodal lymphangioma in an adult: a clinical case report. Cureus. (2023) 15:e50402. 10.7759/cureus.50402 38213354 PMC10783834

[16] Motaganahalli RL Smeds MR Harlander-Locke MP Lawrence PF Fujimura N DeMartino RR A multi-institutional experience in adventitial cystic disease. J Vasc Surg*.* (2017) 65(1):157–61. 10.1016/j.jvs.2016.08.079 27751735

[17] Atamne O Rosenthal E Rubinstein C Rabinovitch Y Sheick-Yousif B. Common femoral adventitial cystic disease in a young female patient. J Vasc Surg Cases Innov Tech*.* (2022) 9(1):101070. 10.1016/j.jvscit.2022.11.008 36718217 PMC9883256

[18] Lim JH Chung BH Kang JH Heo SH Kim DI Kim YW Surgical strategy to reduce the recurrence of adventitial cystic disease after treatment. Vasc Specialist Int*.* (2019) 35(4):217–24. 10.5758/vsi.2019.35.4.217 31915666 PMC6941769

[19] Lun Y Zhang J Jiang H Xu D Sun J Wang S Treatment options for venous cystic adventitial disease: a case report and literature review. Ann Vasc Surg. (2020) 64:413.e1–e4. 10.1016/j.avsg.2019.10.057 31669477

